# Health information priorities for more effective implementation and monitoring of non-communicable disease programs in low- and middle-income countries: lessons from the Pacific

**DOI:** 10.1186/s12916-015-0482-5

**Published:** 2015-09-21

**Authors:** Hebe N. Gouda, Nicola C. Richardson, Robert Beaglehole, Ruth Bonita, Alan D. Lopez

**Affiliations:** School of Public Health, University of Queensland, Brisbane, QLD Australia; World Health Organization, Western Pacific Regional Office, Suva, Fiji; School of Population Health, University of Auckland, Auckland, New Zealand; Melbourne School of Population and Global Health, the University of Melbourne, Melbourne, VIC Australia

**Keywords:** Health information systems, Non-communicable diseases, Monitoring and surveillance

## Abstract

**Background:**

Non-communicable diseases (NCDs) place enormous burdens on individuals and health systems. While there has been significant global progress to guide the development of national NCD monitoring programs, many countries still struggle to adequately establish critical information systems to prioritise NCD control approaches.

**Discussion:**

In this paper, we use the recent experience of the Pacific as a case study to highlight four key lessons about prioritising strategies for health information system development for monitoring NCDs: first, NCD interventions must be chosen strategically, taking into account local disease burden and capacities; second, NCD monitoring efforts must align with those interventions so as to be capable of evaluating progress; third, in order to ensure efficiency and sustainability, NCD monitoring strategies must be integrated into existing health information systems; finally, countries should monitor the implementation of key policies to control food and tobacco industries.

**Summary:**

Prioritising NCD interventions to suit local needs is critical and should be accompanied by careful consideration of the most appropriate and feasible monitoring strategies to track and evaluate progress.

## Background

A United Nations High-Level Meeting in 2011 drew attention to the urgent need to control the global non-communicable disease (NCD) epidemic [1]. However, progress has been very slow [2]. The 2014 global review, which took stock of advances since 2011, recognised the critical importance of effective monitoring and surveillance systems in combating NCDs, but said little about the enormous challenges faced by member states in collecting the relevant data to support NCD control efforts. There has been a systematic failure to invest, lead, and catalyse essential health information system developments to reliably monitor progress with NCD programs and policies. The implication is that countries will not be able to effectively inform and evaluate their intervention strategies for some of the most pressing global public health threats, including tobacco, obesity, sub-optimal blood pressure, and other key determinants of increased cardiovascular disease risk. Well-functioning health information systems, civil registration and vital statistics in particular, are essential for more effectively tackling health challenges, ensuring accountability and providing the essential health intelligence to inform strategies to improve population health [3, 4].

Although age-standardised NCD death rates are decreasing worldwide, deaths due to NCDs make up a growing proportion of all deaths globally [5], including in the Pacific [6]. The disability caused by NCDs places enormous burdens on individuals and health systems, slowing economic development and threatening livelihoods [7, 8]. Decades of research have identified a number of NCD ‘best-buy’ interventions [9]; nevertheless, limited national resources and capacity are likely to mean that countries will need to prioritise their strategic options [2], including efforts to monitor NCDs and to promote accountability.

There has been important progress in guiding national strategies to monitor NCDs. In 2013, the World Health Assembly adopted the ‘Comprehensive Global Monitoring Framework, indicators and targets for the prevention and control of NCDs’ (GMF), including nine targets (an NCD premature mortality reduction target, six risk factor targets, and two health system targets) to be achieved by 2025 with 2010 as the baseline. Twenty five indicators to track progress towards these targets are listed in Table 1. Despite this excellent conceptual basis for comprehensive NCD control, countries may struggle to establish and/or maintain adequate monitoring systems to reliably inform what progress, or not, they are making in controlling NCDs.

**Table 1:** Global monitoring framework targets and indicators

Target	Indicator
1. (a) Reduce premature mortality from NCDs by 25 %	1. Unconditional probability of dying between ages 30 and 70 years from cardiovascular disease (CVD), cancer, diabetes, or chronic respiratory diseases
(b) Cancer morbidity	2. Cancer incidence by type of cancer per 100,000 population
2. At least 10 % relative reduction in the harmful use of alcohol, as appropriate within the national context	3. Total (recorded and unrecorded) alcohol per capita (15+ years old) consumption within a calendar year in litres of pure alcohol, as appropriate within the national context
	4. Age-standardised prevalence of heavy episodic drinking among (adolescents and adults) as appropriate within the national context
	5. Alcohol-related morbidity and mortality among adolescents and adults, as appropriate within the national context
3. 10 % relative reduction in prevalence of insufficient physical activity	6. Age-standardised prevalence of insufficiently active adults aged 18+ years (defined as less than 150 minutes of moderate intensity activity per week or equivalent)
	7. Prevalence of insufficiently physically active adolescents defined as less than 60 minutes of moderate to vigorous intensity activity daily
4. 30 % reduction in mean population intake of salt/sodium	8. Age-standardised mean population intake of salt (sodium chloride) per day in grams in adults aged 18+ years
5. 30 % reduction in prevalence of current tobacco smoking	9. Age-standardised prevalence of current tobacco smoking among persons aged 18+ years
	10. Prevalence of current tobacco use among adolescents
6. Halt the rise in diabetes and obesity	11. Age-standardised prevalence of raised blood glucose/diabetes among adults aged 18+ years (defined as fasting plasma glucose value ≥7.0 mmol/L (126 mg/dL) or on medication for raised blood glucose)
	12. Age-standardised prevalence of overweight and obesity in adults aged 18+ years (defined as body mass index (BMI) ≥25 kg/m for overweight or ≥30 kg/m for obesity)
	13. Prevalence of overweight and obesity in adolescents (defined according to the WHO Growth Reference: overweight – one standard deviation BMI for age and sex, and obese – two standard deviations BMI for age and sex)
7. (a) 25 % relative reduction in the prevalence of raised blood pressure or contain the prevalence of raised blood pressure according to national circumstances	14. Age-standardised prevalence of raised blood pressure among adults aged 18+ years (defined as systolic blood pressure ≥140 mmHg and/or diastolic blood pressure ≥90 mmHg) and mean systolic blood pressure
(b) Cholesterol	15. Age-standardised prevalence of raised total cholesterol among adults aged 18+ years (defined as total cholesterol ≥5.0 mmol/L or 190 mg/dL
(c) Fat intake	16. Age-standardised mean proportion of total energy intake from saturated fatty acids and polyunsaturated fatty acids in adults aged 18+ years
(d) Fruit and vegetable intake	17. Age-standardised prevalence of adult (aged 18+ years) population consuming less than five total servings (400 g) of fruit and vegetables per day
8. At least 50 % of eligible people receive drug therapy and counselling (including glycaemic control) to prevent heart attacks and strokes	18. Proportion of eligible persons (defined as aged 40 years and over with a 10-year cardiovascular risk greater than or equal to 30 % including those with existing CVD) receiving drug therapy and counselling to prevent heart attacks and strokes
9. (a) An 80 % availability of the affordable basic technologies and essential medicines, including generics, required to treat major non-communicable diseases in both public and private facilities	19. Availability and affordability of quality, safe, and efficacious essential non-communicable disease medicines, including generics, and basic technologies in both public and private facilities
(b) Palliative care	20. Access to palliative care assessed by morphine-equivalent consumption of strong opioid analgesics (excluding methadone) per death from cancer
(c) Cervical cancer	21. Proportion of women between the ages of 30 and 49 screened for cervical cancer at least once, or more often, and for lower and higher age groups according to programs and policies
(d) Trans-fat elimination	22. Adoption of national policies that virtually eliminate partially hydrogenated vegetable oils in the food supply and replace with polyunsaturated fatty acids
(e) Marketing foods to children	23. Policies to reduce the impact on children of marketing of foods high in saturated fats, trans-fatty acids, free sugars, or salt
(f) Vaccination against cancer-causing infections	24. Vaccination coverage against hepatitis B virus monitored by number of third doses of Hep-B vaccine (HepB3) administered to infants
	25. Availability, as appropriate, if cost-effective and affordable, of vaccines against human papillomavirus, according to national programmes and policies

Moreover, data requirements for more effective NCD control are only one component of a broader demand on countries for reliable burden of disease data. The recent Ebola outbreak highlighted the tragic vulnerability of populations in the absence of strong health surveillance and health systems. At the same time, the evaluation of national progress towards Millennium Development Goal targets has been hampered by the lack of reliable, timely, and comparable data in most low- and middle-income countries (LMICs) [10]. Negotiations surrounding the new sustainable development agenda have demonstrated that the debate has shifted from one about ‘what works’ to issues of accountability. The new development goals should provide the necessary incentive to strengthen surveillance systems in order to ensure accountability frameworks can be upheld. A recent high-level summit on Measurement and Accountability for Results in Health [11] suggests that the central role of data systems in development is increasingly recognised. In light of these advances, the state of national health information systems and the actions necessary to improve them will hopefully gain greater prominence.

### Epidemiological transition in the Pacific

In this paper, we focus on the pressing need to adequately incorporate NCDs into this evolving health measurement and accountability dialogue and suggest a framework for prioritising monitoring efforts, particularly in resource-poor countries. Over the past two decades, Pacific Island Countries and Territories (PICTs) have seen a rapid increase in NCDs and now suffer from some of the highest burdens due to NCDs in the world [12]. The Global Burden of Disease has estimated that NCDs amongst those PICTs (for which data is available) made up 33.6 % of the total disease burden in 1990, rising to 47.7 % in 2010 [13]. Additionally, obesity levels are some of the highest in the world; prevalence of obesity amongst females is over 75 % in four Pacific countries and between 12.4 % and 53.4 % of people aged between 25 and 64 are affected by diabetes [14]. Furthermore, the prevalence of NCD risk factors, such as insufficient physical activity, tobacco, and heavy alcohol consumption, are exceptionally high in a number of PICT populations, presenting discouraging signs of future burdens to come [14]. PICTs, therefore, need to take urgent action on NCDs. Similar to many other LMICs, PICTs struggle to address the NCD burdens and are severely hampered by weak health information systems and a lack of reliable timely data (Table 2). Despite these challenges and setbacks, PICTs have developed some innovative strategies and collaborations to tackle NCDs. We use the experiences of the PICTs as a case study of how to strategically and feasibly enhance monitoring systems for NCD control through considered choices about what data to collect and how to ensure their quality and relevance. This need is especially urgent in the Pacific given the extraordinary levels of premature adult mortality [15], but the approach we propose could equally inform best practice elsewhere.

**Table 2:** Data sources and availability of relevant data (key population health surveys) from Pacific Island Countries and Territories (2002–2015)^a^

Country	Years in which a STEPS survey was conducted	Year of Demographic Health Survey	Most recent GSHS	NCD Country Capacity Survey	Availability of mortality estimates for 2012 (Global Status of NCDs 2014 [37])
Cook Islands	2004 and 2014	–	2010	2010	–
Fiji	2002 and 2011	–	2010	2010	yes
Kiribati	2004–06 (and 2015 near completion)	2009	2011	2010	–
Nauru	2004	2007	2011	2010	–
Niue	2011		2010	2010	–
Samoa	2004 and 2013	2009	2011	2010	–
Solomon Islands	2006 and 2015	2007	2011	2010	yes
Papua New Guinea	2007–08	1996 and 2006	2007	2010	yes
Tokelau	2005 and 2015	–	–	–	
Tonga	2004 and 2012	2012	2010	2010	–
Tuvalu	2007	2007	2013	2010	–
Vanuatu	2011	2013	2011	2010	–

GSHS, Global School-based Student Health Survey; NCD(s), Non-communicable disease(s); STEPS, WHO STEPwise Surveillance of NCD risk factors

^a^For the purpose of this paper United States Affiliated Pacific Island countries were not considered

The Pacific is in the midst of a NCD crisis [16], while continuing to deal with residual maternal and child health issues and disease outbreaks. When available, data on NCDs and causes of death in the Pacific are generally outdated or of poor quality (Table 2). Estimates of disease burden in the region, like those that are reported by the Global Burden of Disease, are highly uncertain and insufficiently reliable to monitor changes [17]. PICTs have struggled to develop and maintain health information systems to collect and analyse data and to report on the health of their populations. This is further exacerbated by parallel systems of disease monitoring which, while perhaps meeting development partner requirements, have resulted in duplication of effort and potentially weakened more comprehensive health information system development in countries. Despite recent efforts to rectify these issues, exemplified by the migration of data collection tools for HIV, tuberculosis, and NCD programs in the Solomon Islands into the national health information system [18], there has been little appreciation of the crucial role that reliable and timely health information plays in national disease control strategies. There is a real danger that the challenges posed by the lack of capacity currently accessible within these systems will perpetuate the inertia that plagues NCD control efforts in the region.

## Discussion

Based upon the experience of the Pacific thus far in monitoring and controlling NCDs, we share four key lessons that we believe are broadly relevant for health information systems development so that countries are better prepared to control their NCD epidemics, accelerate health system responses, and report on progress between now and 2025, as called for in the WHO GMF.

### Lesson One: NCD intervention priorities need to be strategically chosen

Achieving all the globally agreed voluntary targets will be impossible for many countries, and it is important that they do not set themselves up for failure. As a key step towards reducing the major NCD burdens in their populations, Pacific Ministers of Health and Finance jointly agreed on a Roadmap for NCDs that set four intervention priorities: tobacco control, policies to reduce the consumption of unhealthy foods and drinks, scaling up NCD interventions in primary healthcare settings, and strengthening the evidence base to assess NCD program investments [6]. As Pacific countries progressively roll out the interventions required and the systems to measure their success, they also have a menu of 30 other areas they might address, according to their local circumstances and needs (see NCD Roadmap [19]). Other countries should likewise consider the ‘best-buys’ in relation to local NCD burdens and context to prioritise actions for controlling and preventing NCDs.

### Lesson Two: NCD monitoring strategies must be aligned with prioritised interventions

**Fig. 1:**
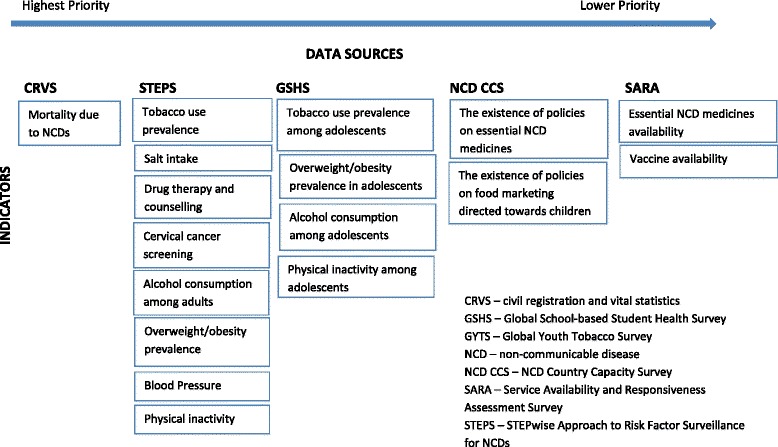
Priority data sources and indicators necessary for monitoring NCDs [Source: Health Information Systems Knowledge Hub Working Paper 33 (22)]

Intervention priorities should dictate the monitoring priorities. Many countries are not able to collect all the data necessary to fulfil the requirements of the NCD framework. Good quality data on a small number of key indicators are likely to be more useful for policy than large amounts of (often unreliable) data that distract from the critical information needs to address stated priorities. To ensure that a country’s health information system is capable of effectively tracking epidemiological changes, a minimum dataset should be prioritised and collected. In the case of NCDs, this consists of reliable and timely vital registration data to allow continuous monitoring of cause-specific mortality, cross-sectional surveys of population exposure to major risk factors for the leading causes of NCDs – ideally three before 2025 – and periodic documentation of the effective coverage of key NCD interventions [20]. Two data sources are essential if countries are to be able to report progress on NCDs in 2025 – civil registration and vital statistics systems that reliably capture all deaths and include established procedures to document causes of death, including medical certification or, where certification is not available, automated verbal autopsy methods [21]; and the WHO STEPwise Surveillance of NCD risk factors (STEPS) – or equivalent – surveys on risk factor levels and patterns in the population. Based upon priority interventions and the feasibility of collecting relevant indicators in PICTs, Fig. 1 presents a list of prioritised data sources and indicators (further details on how this prioritisation was conducted can be found in a the Health Information Systems Knowledge Hub Working Paper 33 [22]). Strengthening civil registration and vital statistics systems for registering deaths and correctly certifying cause of death is the only strategy that can provide reliable information on changes in NCD mortality patterns [4]. This will require intensive training for physicians in correct procedures for death certification and the wider use of automated verbal autopsies to ascertain cause of death in deaths that occur outside hospital settings. Through the efforts of the Brisbane Accord Group and the Pacific Vital Statistics Action Plan , there is growing recognition of the importance of vital statistics among PICTs [23], with small but notable improvements already apparent in some national vital registration systems [24]; for example, Fiji has developed and implemented a comprehensive training program to improve medical certification of death, including routine data quality audits, and Niue has produced its first vital statistics report in 20 years.

If STEPS surveys are undertaken, with the addition of a module on salt and a few additional questions related to treatment, countries will be able to report on five of the risk factor targets, including self-reported smoking, harmful use of alcohol, salt consumption, raised blood pressure, and physical inactivity, as well as one of the agreed health system targets, namely treatment with combination drugs for those at highest absolute risk of heart attack and stroke. Ideally, all PICTs would have conducted at least two (preferably three, including the baseline survey) STEPS surveys before reporting on progress in 2025 [5]. As STEPS was adopted by Pacific countries relatively early, almost all countries have already established baseline measures of risk factor prevalence; four countries have completed a second round. Commitment to repeating cross sectional surveys using the same methodology is key. Greater use of electronic data collection and data analyses packages since 2009 has overcome earlier challenges that impeded rapid data analyses and reporting. Selectively including the objectively measured Step 3 (physical measures and blood collection) will reduce both cost and complexity. A data analysis and reporting team is now coordinated by WHO; together with technical input from the Monitoring Alliance for NCD Action (MANA), these developments could provide a platform to assist countries to improve their data systems to inform policy action on NCDs, and support tracking progress and reporting against the priority targets set by each country. The proposed NCD Countdown 2025 template [25] and the Pacific MANA dashboard [26] currently under development could be used to summarise progress with the overall mortality target and levels of risk factor prevalence. Other countries and regions where the capacity of the existing health information systems is limited could consider similar strategies to prioritising monitoring efforts.

### Lesson Three: NCD monitoring strategies should be integrated into existing health information systems and coordinated with existing data strengthening efforts, such as for civil registration and vital statistics under the Brisbane Accord Group

Monitoring and surveillance are resource-intensive activities. Leaders in the Pacific have expressed concerns about the large number of goals, targets, and indicators emerging from discussions on the sustainable development goals [27]; though the targets are set 5 years beyond the 25 by 25 goals, proposals for sustainable development goal indicators thus far are aligned with GMF indicators. Ensuring that these targets and indicators are integrated into existing national systems can help relieve some of the burden associated with monitoring and surveillance. The civil registration and vital statistics system is the backbone of a national health information system. It must be fit for purpose and its sustainability must be ensured. In the Pacific, the Brisbane Accord Group has provided a valuable resource to the region offering a collaborative platform for coordinating the work of partner agencies and providing strategic and technical support to improve vital statistics, including data routinely collected from health facilities. The Brisbane Accord Group has helped develop and support country-led Civil Registration and Vital Statistics Committees, which have members from health, statistics, and registration – ensuring links between all systems and departments (including Information Technology). An example of action to strengthening data collection at the health facility level is provided by ‘PEN (Package of Essential NCD Interventions) Fa’a Samoa’; an award-winning community-based programme aimed at early detection of NCDs in select villages in Samoa. As part of the initiative, community registration forms collecting data on NCD risk factors are managed by local Village Women’s Committees and the programme works with local hospital hospitals to improve links between outreach/primary services and to strengthening medical records.

Many challenges still remain in PICTs, but steady progress is evident. Recent initiatives intended to intensify technical assistance to countries to strengthen civil registration and vital statistics systems ought to help accelerate this trend [28].

### Lesson Four: Develop strategies to monitor the implementation of selected policies to regulate the food and tobacco industries

Access to unhealthy foods and products and the trade agreements that facilitate their consumption and affordability are increasingly recognised as important drivers of the NCD epidemic [29, 30], acting in a similar fashion to the promotion of tobacco use. PICTs have been particularly active in adopting tobacco regulation and control efforts. Nine Pacific countries have taken action to implement comprehensive tobacco control through increasing the tobacco tax, an extremely effective tobacco control measure [31], within the last three years. Ministers of Health have called for a Tobacco Free Pacific by 2025, a key step in providing the public health leadership and resources required to drastically reduce tobacco use. Furthermore, nutrition labels are now mandatory in six countries, salt targets have already been adopted in five and twelve countries have introduced a sugar tax [32]; other PICTs are set to follow as capacity expands. The NCD Country Capacity Survey is a first step in tracking the uptake of these policies. In taking trade regulation seriously, the PICTs are in a position to potentially set an example for many other LMICs. Monitoring and disseminating information on the implementation and eventually the impact of these key public health actions will further support national NCD control efforts.

### Global progress

Though most countries have adopted the voluntary targets of the GMF for NCDs, there is still only limited progress in implementing the priority interventions, except on tobacco control. Countries should consider their own NCD priorities and capacities before adapting global monitoring strategies to their own context. We have focused on the PICTs since they have begun this process, but they are not alone. India, for instance, recently unveiled a National Multi-sectoral Action Plan in which the GMF has been adapted and put into action; recognising the burden due to indoor air pollution, India has added a tenth target to those proposed by the GMF [33]. The Caribbean Islands have also recently conducted a data gap analysis and considered their priorities in terms of interventions and policy actions [34]. Finally, the US-affiliated Pacific Islands have developed a detailed monitoring plan based upon existing data sources [35].

### Summary

Better data is the first step in the development and strengthening of mechanisms to identify and track public health challenges within countries and globally, and to be able to hold governments and industries accountable for actions and inactions. Problems with data from the Pacific region are reflected in the uncertainty in Global Burden of Disease estimated for the Oceania region, but nascent efforts to strengthen health information systems in PICTs are evident and laudable. Nonetheless, efforts need to be intensified through more effective leadership, technical assistance, and resources. Though progress in NCD control has been slow, we have outlined here the valuable lessons that the Pacific experience thus far can offer other LMICs who are, or soon will be, struggling to address high NCD burdens, and who too must deal with scarce resources and a limited health information system capacity. Prioritising NCD interventions to suit local needs is critical, and should be accompanied by careful consideration of the most appropriate and feasible monitoring strategies to track and evaluate progress.

Despite encouraging signs emerging from the global development community, led by the WHO, there is widespread and alarming ignorance of the likely scale of the NCD crisis worldwide, including the Pacific. Regrettably, NCDs still fail to garner the same international attention as Maternal and Child Health, perhaps because many countries, like many of the PICTs, are overcome by the challenge. Over the last few decades, child mortality has fallen substantially in the Pacific, yet the continuing rise in NCDs is largely ignored, despite vociferous calls to action [36]. Indeed, global health priorities are not an ‘either/or’ proposition; both the massive premature mortality due to NCDs and the residual Maternal and Child Health agenda should be at the forefront of global health action in our quest for a healthier world. The post-2015 agenda provides us the opportunity to renew our commitment to this vision while also giving us the unique opportunity to bring together multiple sectors to address difficult health problems, like NCDs, effectively and sustainably. Strong leadership, concerted efforts to strengthen technical capacity and improved country-level organisation and resources are essential if we are to make demonstrable progress in the monitoring and control of key health challenges, in PICTs, and elsewhere.

## Abbreviations

GMF: Global Monitoring Framework
LMICs: Low- and middle-income countries
NCD: Non-communicable diseases
Pacific MANA: Pacific Monitoring Alliance for NCD Action
PICTs: Pacific Island Countries and Territories
STEPS: WHO STEPwise Surveillance of NCD risk factors
