# Erratum to: Health information priorities for more effective implementation and monitoring of non-communicable disease programs in low- and middle-income countries: lessons from the Pacific

**DOI:** 10.1186/s12916-015-0506-1

**Published:** 2015-10-20

**Authors:** Hebe N. Gouda, Nicola C. Richards, Robert Beaglehole, Ruth Bonita, Alan D. Lopez

**Affiliations:** School of Public Health, University of Queensland, Brisbane, QLD Australia; World Health Organization, Western Pacific Regional Office, Suva, Fiji; School of Population Health, University of Auckland, Auckland, New Zealand; Melbourne School of Population and Global Health, the University of Melbourne, Melbourne, VIC Australia

## Erratum

After publication of the original article [1] it was noted that the author Nicola C. Richards’ surname was spelled incorrectly. When published initially it was spelled ‘Richardson’. This has now been corrected in the original article.
